# Pain Management Frameworks and Critical Care Nursing Competencies: An Evidence-Informed Integrative Review

**DOI:** 10.7759/cureus.105039

**Published:** 2026-03-11

**Authors:** Abeer Almutairi, Faridah Mohd Said

**Affiliations:** 1 Nursing, Lincoln University, Petaling Jaya, MYS

**Keywords:** clinical competency, critical care nursing, multimodal analgesia, pain assessment tools, pain management

## Abstract

Pain is a prevalent and complex challenge in intensive care units (ICUs). Although structured pain management frameworks, such as validated behavioral assessment tools and multimodal analgesia protocols, are widely recommended, implementation variability persists. Emerging evidence suggests that nursing competency and organizational context influence the fidelity of these frameworks. This review synthesized contemporary literature examining ICU pain management frameworks and the nursing competencies required for effective implementation. An evidence-informed integrative review was conducted using a structured search of PubMed, CINAHL, EMBASE, Cochrane Library, and PsycINFO for studies published between January 2020 and March 2025. Empirical, validation, and guideline-based studies conducted in adult ICUs were included. The literature search yielded 10 studies, and data were extracted and thematically synthesized to identify pain management frameworks, nursing competency domains, and contextual determinants influencing practice. Findings indicate that ICU pain management is structured around validated behavioral assessment tools, including the Behavioral Pain Scale and the Critical-Care Pain Observation Tool, multimodal analgesia strategies, and protocol-driven monitoring. However, evidence demonstrates variability in nurses’ pharmacological knowledge, assessment proficiency, clinical decision-making behaviors, and attitudes toward analgesia. Organizational factors, including workload pressures, staffing and patient factors, and institutional protocol reinforcement, moderate implementation consistency. The synthesis informed a three-level conceptual model linking frameworks, competency domains, and organizational determinants. Effective ICU pain management requires alignment between standardized frameworks, multidimensional nursing competencies, and supportive organizational systems. Integrated competency development and protocol reinforcement may enhance consistency and improve patient outcomes in critical care settings.

## Introduction and background

Pain management remains a paramount yet frequently inadequate clinical issue in critical care environments. Studies indicate that patients in intensive care units (ICUs) commonly endure moderate-to-severe pain arising from surgical interventions, trauma, invasive procedures, mechanical ventilation, and pathological conditions [1,2]. Ineffective pain mitigation is associated with adverse outcomes, including prolonged mechanical ventilation, disturbances in cognitive status such as delirium, alterations in hemodynamics, extended ICU stays, and potential long-term psychological impacts [2,3]. Notwithstanding advances in analgesic medications and validated pain assessment tools, inconsistencies in pain management protocols remain evident in critical care practice.

Pain assessment in critically ill, sedated adult patients is challenging because many are intubated or cognitively impaired due to severe illness and associated treatments. In such cases, it is recommended that nurses use behavioral assessment tools to evaluate pain, such as the Behavioral Pain Scale (BPS) or the Critical-Care Pain Observation Tool (CPOT) [4,5]. The BPS evaluates pain in mechanically ventilated patients through three behavioral indicators, namely, facial expression, upper limb movements, and compliance with mechanical ventilation. Each indicator is scored on a scale ranging from 1 to 4, with the total score (3-12) reflecting increasing levels of pain intensity. Similarly, the CPOT assesses four behavioral domains, namely, facial expression, body movements, muscle tension, and either compliance with ventilation (for intubated patients) or vocalization (for non-intubated patients). Each domain is scored from 0 to 2, yielding a total score between 0 and 8, with higher scores indicating greater pain [4,5]. These tools are incorporated into international clinical practice guidelines for pain assessment and management in intensive care settings, including the Pain, Agitation, and Delirium (PAD) guidelines originally published by Devlin et al. [3]. However, the effective use of these tools requires interpretive skills, accurate observation, and clinical contextual reasoning.

In the ICU, pain management is increasingly guided by the principles of multimodal analgesia that combine the use of opioid and non-opioid medications with adjuvant drugs as well as non-pharmacological interventions such as positioning, massage, relaxation techniques, and cold application [6,7]. While a multimodal approach can achieve superior analgesia with fewer adverse effects of opioids, it requires a deep understanding of medication regimens, careful monitoring, and ongoing evaluation.

Studies have reported the knowledge, attitudes, and practice gap among nurses regarding pain management in ICUs. According to a scoping review, knowledge of pain among nurses and the use of assessment tools in healthcare settings have been reported as inadequate [8]. A cross-sectional study among ICU nurses reported moderate knowledge and practice regarding documentation and the use of behavioral scales [9]. Two other studies reported differences in pain management attitudes and practices among critical care nurses across countries [10,11]. These findings suggest that competency limitations may contribute to inconsistent implementation of established pain management frameworks.

Nursing competency in critical care extends beyond medication administration to encompass integrated knowledge, clinical judgment, ethical advocacy, communication, and contextual awareness [12,13]. Effective pain management requires the ability to differentiate pain from agitation or delirium, titrate analgesics safely, recognize cultural influences on pain expression, and advocate for appropriate interventions within interdisciplinary teams. Moreover, organizational determinants, including workload, staffing patterns, protocol availability, and access to continuing education, significantly influence the translation of competency into practice [14].

While several studies have reported educational strategies to increase nurses’ knowledge in pain management, few have considered how pain management frameworks align with nursing competencies. Given the complexity of pain and the intensity of care in the ICU, it is important to understand the potential relationships among these frameworks and competencies to enhance care consistency and improve patient outcomes. In this integrative review of the literature from 2020 to2025, we seek to evaluate frameworks of pain management in critical care, nurses’ competencies in critical care, and organizational influences on critical care nursing competencies. This review aims to combine the findings of previous empirical research with expert perspectives to understand the relationship between pain management frameworks and the competencies of nurses practicing in critical care.

## Review

Methodology

An evidence-informed integrative review of the contemporary literature regarding pain management strategies in intensive care and the nursing competencies relating to effective pain management was undertaken as part of this study. The integrative review methodology has been reported as particularly suited to including diverse empirical study designs, such as quantitative, qualitative, and mixed-methods research, within a single review to provide a more comprehensive understanding of complex clinical issues [15]. Unlike systematic reviews conducted by meta-analysis, integrative reviews focus on thematic and conceptual synthesis rather than statistical synthesis. Although this study was conducted as an integrative review rather than a formal systematic review with meta-analysis, the study selection and reporting process were informed by the Preferred Reporting Items for Systematic Reviews and Meta-Analyses (PRISMA) 2020 statement to enhance transparency and reproducibility of screening procedures [16].

An evidence search was conducted in the peer-reviewed literature published between January 2020 and December 2025 to identify relevant studies. This timeline was selected to reflect current trends in research on multimodal analgesia, pain management procedures in ICUs, and nursing competency development. The literature published before 2020 was consulted to contextualize the review; only studies published between 2020 and 2025 were included in the formal synthesis. The databases utilized for the search were PubMed, CINAHL, EMBASE, Cochrane Library, and PsycINFO. Search terms were developed using combinations of controlled vocabulary and free-text keywords. These included “critical care nurses” or “intensive care unit nurses,” “pain management” or “pain assessment,” “multimodal analgesia,” “behavioral pain scale” or “Critical-Care Pain Observation Tool,” “competency” or “clinical competence,” and “critical thinking” or “clinical judgment.” Boolean operators (AND/OR) were applied to refine the search strategy and ensure an appropriate combination of concepts.

Inclusion and Exclusion Criteria

The inclusion and exclusion criteria are shown in Table 1.

**Table 1:** Inclusion and exclusion criteria. ICU = intensive care unit

Inclusion criteria	Exclusion criteria
Examined pain management practices, frameworks, or protocols in adult critical care settings	Focused exclusively on non-critical care settings
Involved ICU or critical care nurses as primary participants	Examined only physicians or other healthcare professionals without nurse-specific analysis
Assessed nursing competency, knowledge, attitudes, behaviors, or clinical decision-making related to pain management	Editorials, commentaries, conference abstracts, or non-empirical publications
Empirical studies (quantitative, qualitative, or mixed-methods), validation studies, and relevant review-based literature	Addressed pediatric-only populations
Studies published in English between 2020 and 2025	

Study Selection Process

Records identified through the database search were screened according to predefined inclusion and exclusion criteria. After removal of duplicates, titles and abstracts were assessed for relevance, followed by full-text review of potentially eligible studies. Articles meeting the eligibility criteria were included in the final synthesis.

Data from the included studies were systematically extracted and organized into structured matrices to facilitate comparative analysis. Extracted variables included study design, country and clinical setting, pain management frameworks examined, nursing competency domains assessed, and key findings. This structured approach enabled consistent categorization and comparison across heterogeneous study designs and contexts. A thematic analysis was subsequently conducted to identify recurring patterns and conceptual relationships across studies. In accordance with the integrative review framework proposed by Whittemore and Knafl [15], findings were synthesized into three overarching domains, namely, pain management frameworks utilized in critical care settings, core nursing competency domains associated with framework implementation, and organizational or contextual determinants influencing competency performance.

Themes were iteratively refined through constant comparative analysis to ensure conceptual coherence, internal consistency, and alignment with the stated objectives of the review. Although formal risk-of-bias scoring was not conducted due to the integrative nature of the review, the methodological rigor of included studies was considered during synthesis. Particular attention was paid to study design clarity, the measurement tools used (e.g., validated pain assessment instruments), and the consistency of competency evaluation methods.

**Figure 1:**
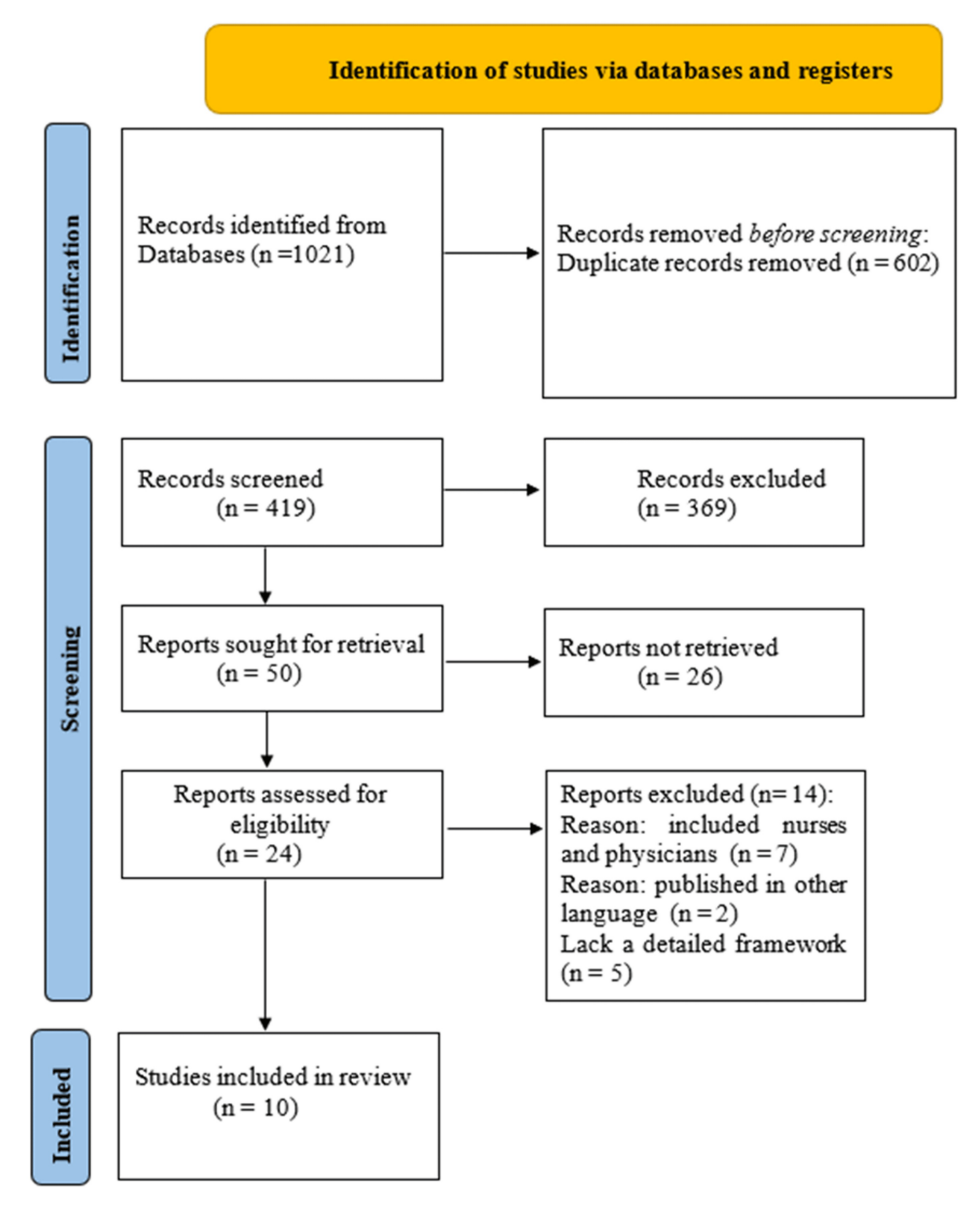
Preferred Reporting Items for Systematic Reviews and Meta-Analyses (PRISMA) flow diagram illustrating the study selection process. The diagram illustrates identification, screening, eligibility assessment, and inclusion of studies in accordance with PRISMA 2020 guidelines.

The study selection process is illustrated in the PRISMA flow diagram (Figure 1). A total of 1,021 records were identified through database searching. After the removal of 602 duplicate records, 419 titles and abstracts were screened for relevance; of these, 369 records were excluded based on predefined inclusion and exclusion criteria. In total, 50 reports were sought for retrieval, of which 26 were not retrieved. Overall, 24 full-text articles were assessed for eligibility. In total, 14 reports were excluded following full-text review due to inclusion of mixed nurse-physician samples without nurse-specific analysis (n = 7), publication in a language other than English (n = 2), or lack of a clearly defined pain management framework (n = 5). Ultimately, 10 studies met the eligibility criteria and were included in the final synthesis.

Results

The body of literature examining pain management competency among nurses in critical care settings reflects considerable methodological diversity, spanning scoping reviews [8], cross-sectional surveys [9-11,14], validation studies, and guideline-based clinical reviews [4-7,17] conducted across varied geographical contexts. Recent investigations by Alzahrani et al. [8] synthesized evidence from multiple countries and hospital settings, highlighting persistent knowledge deficits and inconsistent use of standardized pain assessment tools among nurses, particularly in ICUs. Complementary cross-sectional studies conducted in Pakistan [9], Turkey [10], Greece [11], and Tanzania [14] focused primarily on ICU nurses caring for critically ill adults, underscoring moderate knowledge levels, gaps in pharmacological understanding, documentation inconsistencies, and organizational barriers that impede effective pain management. These studies predominantly assessed competency domains, including knowledge, attitudes, clinical behaviors, decision-making, and perceived institutional constraints.

In contrast, several validation and guideline-oriented reviews emphasized the structural and instrumental dimensions of pain management practice. For instance, Rech et al. [17] examined contemporary multimodal and non-opioid analgesic treatment strategies and emphasized the importance of structured, protocol-driven approaches to acute pain management in clinical settings. Similarly, validation studies by Wojnar-Gruszka et al. [4] and Zhai et al. [5] confirmed the reliability and validity of behavioral pain assessment instruments, such as the BPS and the CPOT, for mechanically ventilated and non-communicative ICU patients. Clinical reviews by Nordness et al. [6] and Wheeler et al. [7] further extended the scope of inquiry to multimodal analgesia and opioid-sparing strategies, emphasizing integration of pharmacological and non-pharmacological interventions. Collectively, these studies constitute a global, methodologically heterogeneous evidence base that examines pain management competency across cognitive, behavioral, and system-level domains in critical care practice (Table 2).

**Table 2:** Characteristics of included studies. ICU = intensive care unit; BPS = Behavioral Pain Scale; CPOT = Critical-Care Pain Observation Tool

Author(s) and year	Study design	Country and setting	Sample characteristics	Pain management frameworks examined	Competency domains assessed	Key findings
Wojnar-Gruszka et al. [4] (2022)	Validation/Clinical review study	ICU settings	Non-communicative ICU patients	BPS, CPOT	Assessment accuracy	Supported the reliability of BPS and CPOT in mechanically ventilated patients
Zhai et al. [5] (2020)	Validation/Clinical review study	ICU settings	Mechanically ventilated adult ICU patients	CPOT	Assessment validity	Confirmed CPOT as a reliable instrument for non-verbal ICU patients
Nordness et al. [6] (2021)	Clinical review	ICU settings	Critically ill adults	Multimodal analgesia; non-pharmacological interventions	Clinical application	Emphasized integration of pharmacological and non-pharmacological strategies
Wheeler et al. [7] (2020)	Clinical review	Hospital settings	Adult patients	Adjuvant analgesics; multimodal pain therapy	Pharmacological knowledge	Highlighted the importance of multimodal analgesia and opioid-sparing strategies
Alzahrani et al. [8] (2025)	Scoping review	Multiple countries; hospital settings	Nurses across clinical settings, including ICUs	Pain assessment tools; general pain management practices	Knowledge; attitudes; tool utilization	Identified persistent knowledge gaps and inconsistent use of standardized pain assessment tools
Shabbir et al. [9] (2025)	Cross-sectional survey	Pakistan; tertiary hospital ICU	ICU nurses caring for critically ill adults	Pain assessment tools; documentation practices	Knowledge; clinical practice behaviors	Moderate knowledge levels; inconsistent documentation and variable tool use
Kara and Çamlı [10] (2025)	Descriptive cross-sectional study	Turkey; multicenter ICU settings	Intensive care nurses	Behavioral pain scales (e.g., BPS/CPOT); pain management behaviors	Knowledge; clinical behavior; decision-making	Nurses reported awareness of tools, but inconsistent behavioral application in practice
Papanikita and Pavlatou [11] (2025)	Cross-sectional survey	Greece; adult ICUs	Registered nurses in the ICU	Pain management practices; attitudes toward analgesia	Knowledge; attitudes	A lack of knowledge among nursing staff regarding pharmacology was found
Ghorbani [14] (2025)	Cross-sectional study	Tanzania; ICU settings	ICU nurses	Pain management barriers within the ICU	Organizational factors; perceived barriers	Nurse-related barriers to pain management mostly involve staff and patients. Improving pain management needs training, assessment tools, and cultural considerations
Rech et al. [17] (2022)	Clinical review	International, Acute care settings	ICU populations (adult)	Multimodal and non-opioid analgesic treatment strategies	Pharmacological strategies; protocol-based pain management	Emphasized multimodal analgesia and structured approaches to optimize pain control while reducing opioid reliance

Thematic synthesis

The thematic synthesis was derived from the empirical, validation, and review-based studies summarized in Table 2. Analysis of the included literature generated three interrelated domains, namely, structured pain management frameworks in critical care; core nursing competency domains associated with pain management implementation; and organizational determinants influencing competency performance.

Theme 1: Structured Pain Management Frameworks in Critical Care

Validation studies support the reliability and clinical utility of behavioral pain assessment tools for use in non-communicative ICU patients. Both the BPS and CPOT provide a structured and reproducible assessment of pain intensity in mechanically ventilated patients [4,5], and are components of current pain management paradigms in ICUs. Despite their strong psychometric validation and endorsement in international clinical guidelines, translating these tools into routine clinical practice remains challenging. Empirical studies indicate that although awareness of these tools is widespread among ICU nurses, practical application and documentation consistency vary. Cross-sectional investigations report moderate levels of knowledge and inconsistent use of behavioral scales in routine practice [9,10]. This gap between validated tool effectiveness and inconsistent bedside implementation highlights the influence of contextual factors such as training adequacy, clinical workload, institutional protocols, and organizational support in determining whether evidence-based assessment tools are consistently applied in ICU practice. These findings suggest that tool availability alone does not ensure uniform implementation.

Clinical review and guideline-based studies highlight the importance of multimodal analgesia as a foundation for contemporary ICU pain management [6,7]. These approaches combine opioid and non-opioid medications with adjuvants and selected non-pharmacological interventions to maximize analgesic efficacy while minimizing adverse effects. Structured approaches emphasizing multimodal and non-opioid analgesic strategies have been associated with improved pain management practices [17]. The literature, therefore, reflects a shift from isolated pharmacologic treatment toward structured, protocol-driven systems of care.

Theme 2: Core Nursing Competency Domains

Across empirical studies, several competency domains were repeatedly assessed in relation to ICU pain management. Multiple cross-sectional surveys evaluated nurses’ knowledge and attitudes regarding pain assessment and analgesic administration. Findings consistently demonstrate moderate levels of knowledge, with identified gaps in pharmacological understanding and variability in opioid-related attitudes [8,9,11]. These knowledge disparities may influence the consistency of pain framework implementation.

While the use of tools such as the BPS and CPOT is supported in the literature, their use requires skill in interpreting behaviors. Studies report inconsistencies in the documentation and use of behavioral pain scales, suggesting that their effective use may depend on training, experience, and context [9,10].

Pain management in the ICU is a dynamic process that requires nurses to make clinical judgments. A study on the practice behaviors of ICU nurses in pain management revealed variability in analgesic titration and evaluation of patient responses, suggesting possible differences in decision-making skills among nurses [10]. This underscores the importance of not only knowledge but also clinical reasoning skills in practice.

Theme 3: Organizational and Contextual Determinants

Poor pain management was associated with perceived organizational barriers such as workload, inadequate nurse staffing, and lack of support from the organization [14]. These factors may limit nurses’ opportunities to act within the framework of pain management. The guideline studies demonstrated that having a structured protocol within the organization supported consistent monitoring and reduced practice variability [3]. Having protocols in place and enacted appeared to moderate the competency performance by providing a standardized process for decision-making.

Integrated Interpretation

**Figure 2:**
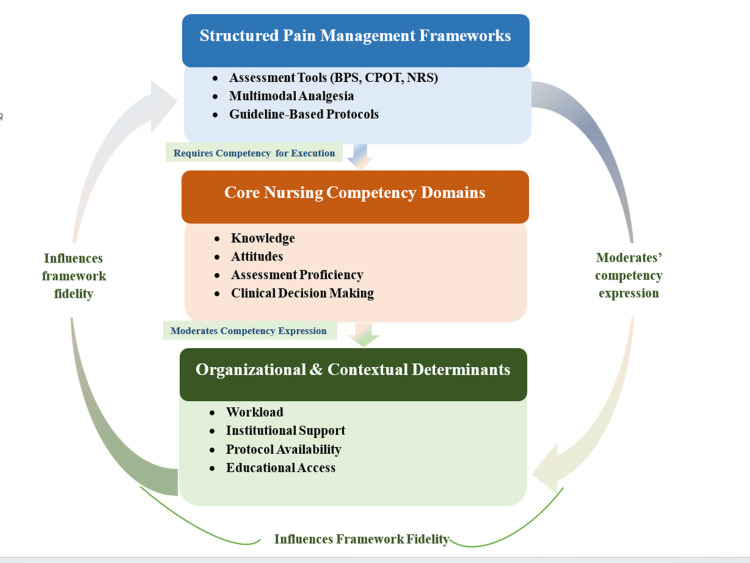
Conceptual model of pain management framework implementation in critical care. The figure was created by the authors using Microsoft Word. BPS = Behavioral Pain Scale; CPOT = Critical-Care Pain Observation Tool; NRS = Numerical Rating Scale

The data in Table 2 indicate that validated pain assessment scales and multimodal analgesic principles underpin the current ICU pain management protocols. However, studies have shown that nurses’ levels of knowledge, skill in assessing behavior, and practice patterns vary. Organizational issues have also been noted to influence the extent to which protocols are implemented in practice. These findings collectively support the conceptual proposition that pain management frameworks and nursing competency domains are interdependent and shaped by contextual determinants within critical care environments (Figure 2).

Discussion

This integrative review has summarized current evidence on the pain management system in critical care and nursing competencies for its effective implementation. The findings presented in Table 2 and Figure 2 demonstrate that the system of pain management, nursing competencies, and organizational factors are interconnected elements rather than separate entities.

At the first level of Figure 2, evidence from the literature supports the presence of frameworks such as behavioral pain assessment tools (BPS and CPOT) [4,5], multimodal analgesia strategies [6,7,17], and structured monitoring recommendations consistent with established intensive care guidelines [3]. These provide a framework for assessing and treating pain in non-communicative patients in the ICU. Despite the presence of these frameworks, studies indicate variability in the use and documentation of behavioral tools [9,10]. This supports the model’s argument that having the framework alone is not sufficient when professionals’ capacities are inadequate.

The second level of Figure 2 represents the multiple competencies required to implement these frameworks. Competency domains, including knowledge of pharmacology, pain assessment, clinical practice, and attitude toward pain management, were addressed by several studies [8,9,11], in which average knowledge and attitudes were moderate and ranged from low to variable. These findings suggest that the level of BPS, CPOT, and multimodal analgesic competencies of the nurse in pain management may contribute to variability in pain management implementation. BPS or CPOT scoring requires interpretation and assessment competency, and multimodal analgesia requires knowledge of pharmacology and clinical decision-making competencies. The second level of Figure 2 represents the cognitive and behavioral competencies required to translate the frameworks presented in the first level into effective clinical practice.

The third level of Figure 2 depicts the moderating effect of the organizational context. Studies identified structural factors, such as workload, nurse-to-patient ratios, staff-related and patient-related factors, and organizational support, as both barriers and facilitators to effective pain management [3,14]. Even when nurses possess adequate knowledge and skills, environmental factors may hinder consistent protocol implementation. This layer of context recognizes that the expression of competence may be influenced by conditions beyond the ability of the individual.

Taken together, Figure 2 conceptualizes ICU pain management as a dynamic interaction between structural frameworks, professional competency, and organizational environment. The findings imply that improvement initiatives implemented at only one of these levels (e.g., new assessment protocols or one-off training seminars) are likely insufficient to create sustainable change in ICU pain management. Instead, a coordinated approach to competency development alongside structural and institutional support may be necessary. From a clinical perspective, this model suggests that the quality of pain management depends on the congruence between competent nursing performance and effective standardized protocols. Training programs could therefore incorporate behavioral assessment, drug reasoning, and decision-making scenarios as complements or alternatives to pure knowledge-based approaches. Stronger support for guideline adherence and lightened workloads may similarly reinforce framework consistency at the institutional level.

Collectively, the integration illustrated in Figure 2 provides a foundation for examining and improving pain management processes in the ICU. The explicit connections between frameworks, competencies, and contextual factors promote movement beyond the isolated examination of educational or process interventions to a more holistic and systemic view of pain management in critical care.

Implications for practice, education, and policy

The three levels of the structure presented in Figure 2 indicate that, to improve pain management in the ICU, interventions may be necessary at the structural, professional, and organizational levels. At the structural level, the regular use of validated tools for behavioral assessment, such as the BPS and CPOT, should be promoted through clinical audit and monitoring of documentation. ICU management cannot rely solely on implementing a standardized tool; they must ensure it is used consistently on a day-to-day basis. At the competency level, education needs to move beyond theoretical and applied clinical reasoning, behavioral assessment, and pharmacotherapeutic training. As studies indicate moderate knowledge and varying use of behavioral tools [8,9], competency-related training should focus on reinforcing clinical application and skills, more so than knowledge.

At the organizational level, healthcare institutions should evaluate staffing adequacy, workload distribution, and protocol accessibility. Organizational barriers such as staffing shortages and high patient acuity may undermine even well-developed competencies [14]. Therefore, institutional reinforcement of structured pain protocols and supportive clinical environments is essential for sustainable improvement.

The findings support a competency-based educational approach tailored specifically to ICU pain management. A competency-based educational approach tailored to intensive care settings should incorporate structured behavioral pain assessment training, opioid stewardship principles, multimodal analgesia reasoning, and scenario-based clinical judgment development. Simulation-based methodologies may enhance experiential learning and improve translation of knowledge into practice. Simulation-based learning may be particularly effective in strengthening applied decision-making skills within complex ICU scenarios. Additionally, structured continuing professional development programs may reduce variability in knowledge and practice behaviors identified across international settings [11].

At the policy level, ICU pain management competency may be considered a quality-of-care indicator. Healthcare systems should integrate compliance with standardized pain assessment into quality monitoring frameworks. Furthermore, institutional policies that support continuing education, reinforce adherence to protocols, and promote interdisciplinary collaboration may enhance the implementation fidelity of pain management frameworks.

Limitations

Several potential limitations should be noted regarding this review. First, although a search strategy with structured evidence identification was employed, this was designed as an integrative rather than a formal systematic review with meta-analysis. Thus, results were not quantitatively aggregated, and a formal risk-of-bias assessment was not performed. Second, differences in study designs, measurement instruments, and outcome reporting among the included studies may limit comparability. Most of the cross-sectional survey designs limit inferences about the impact of competency domains on pain management outcomes. Third, only studies published in English were included, potentially limiting the representation of non-English-speaking ICUs. In addition, differences in healthcare systems and cultures may affect the generalizability of the findings. Finally, the conceptual model presented in Figure 2 is based on a thematic synthesis and has not been empirically validated. Further research should evaluate this framework in longitudinal or interventional designs.

## Conclusions

This integrative review highlights that effective pain management in the ICU depends not only on the availability of validated assessment tools and multimodal analgesic strategies but also on the alignment between structured clinical systems, multidimensional nursing competencies, and supportive organizational environments. Although standardized frameworks for pain assessment and treatment are well established, variability in knowledge, assessment proficiency, clinical reasoning, and contextual constraints continues to influence implementation consistency. From a clinical practice perspective, these findings underscore the need for ongoing training of ICU nurses in behavioral pain assessment, improved competency in the use of validated tools such as the BPS and CPOT, and strengthened clinical reasoning in analgesic decision-making. Furthermore, healthcare institutions should support consistent pain management practices through standardized protocols, continuing professional education, and organizational systems that facilitate the routine application of evidence-based pain assessment frameworks in critical care settings. Improving pain management in critical care, therefore, requires coordinated strategies that strengthen competency development while reinforcing institutional structures that support consistent protocol application. A systems-oriented perspective that integrates clinical frameworks, professional capability, and organizational support may offer a more sustainable approach to enhancing pain management quality in intensive care settings. Future research should empirically evaluate integrated competency-based models to determine their impact on implementation fidelity and patient outcomes.
